# Supplementary material for: Chemical Defense against Herbivory in the Brown Marine Macroalga Padina gymnospora Could Be Attributed to a New Hydrocarbon Compound
Source: Plants (Basel). 2023 Feb 28;12(5):1073. doi: 10.3390/plants12051073 (PMC10005330; doi:10.3390/plants12051073)
Supplement: Supplementary file 1 [file plants-12-01073-s001.zip › plants-2079611-supplementary.pdf]

Supplementary

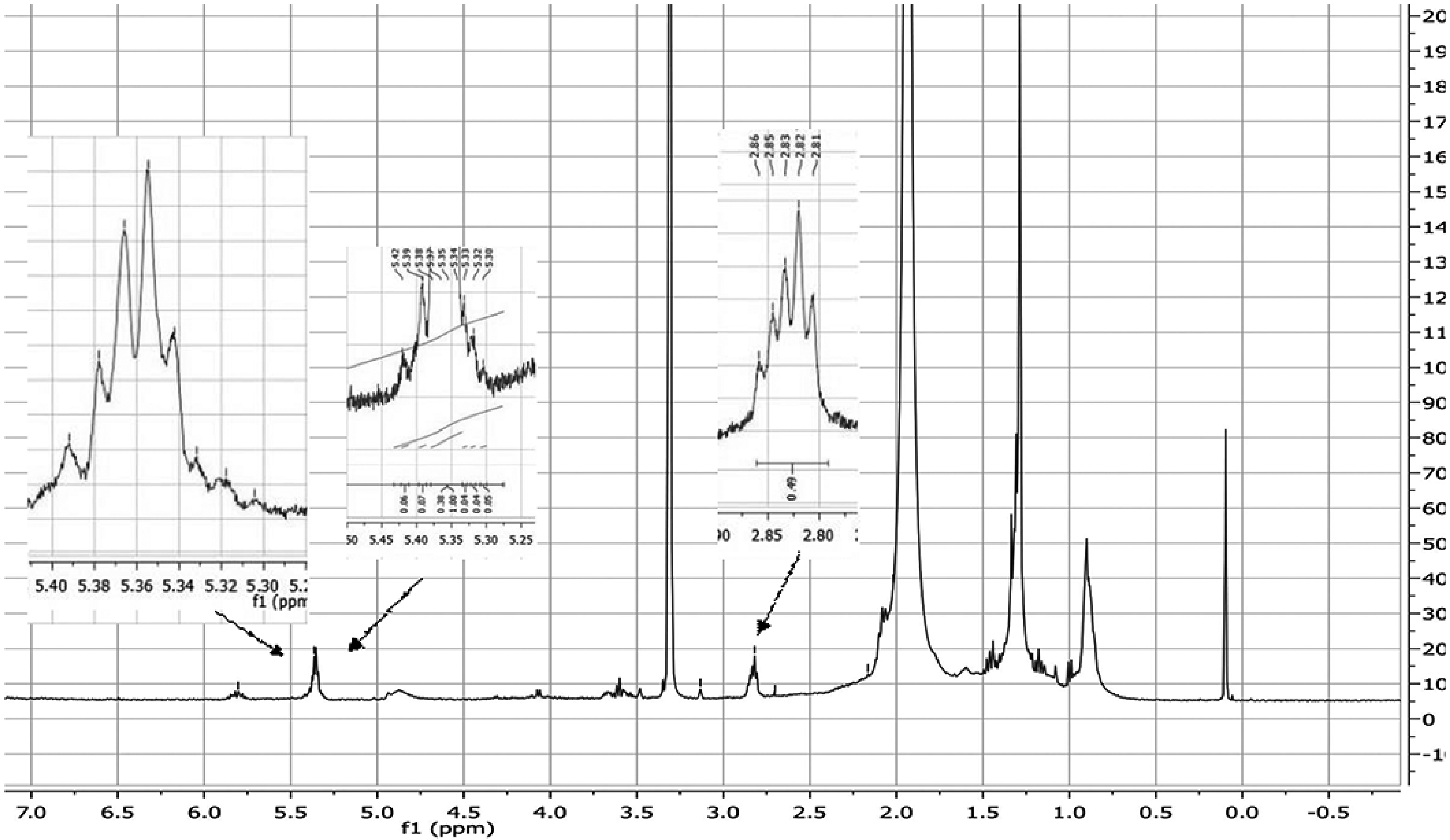

Figure S1 - Nuclear Magnetic Resonance spectral data (ppm) from *P*. *gymnospora* FA3 obtained in deuterated methanol (CD_3OD). Methanol signal at 3.31 ppm.

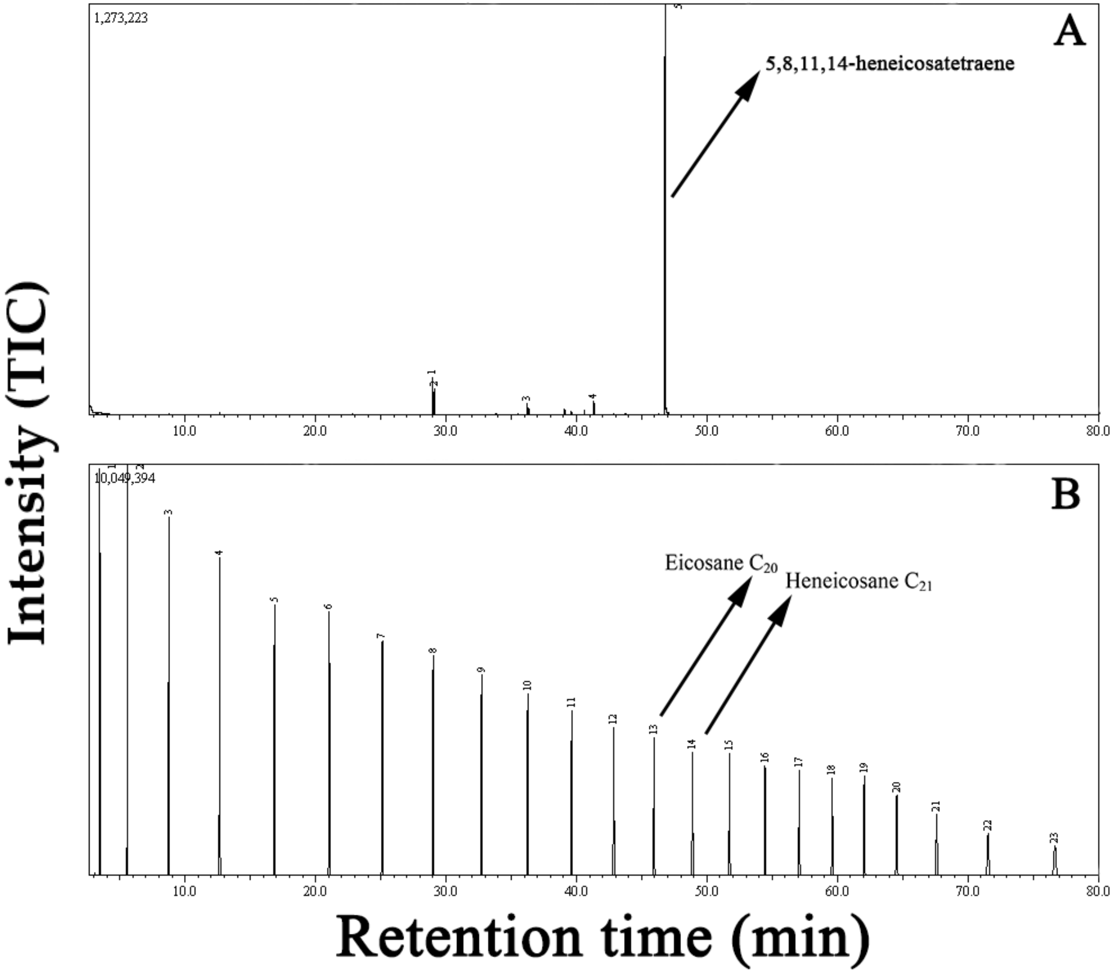

Figure S2- Gas Chromatography coupled to Mass Spectrometry (GC/MS) analysis without esterification or derivatization of FA3 from *P. gymnospora*. **(A)** Arrow shows the peak for the new compound 5*Z*,8*Z*,11*Z*,14*Z*-heneicosatetraene. (B) Chromatogram from the hydrocarbons

standards, highlighting the peaks (arrow) that eluted before eicosane (C_{20}) and after heneicosane (C_{21}). Retention times were used to calculate the retention index for 5*Z*,8*Z*,11*Z*,14*Z*-heneicosatetraene (RI = 2029).

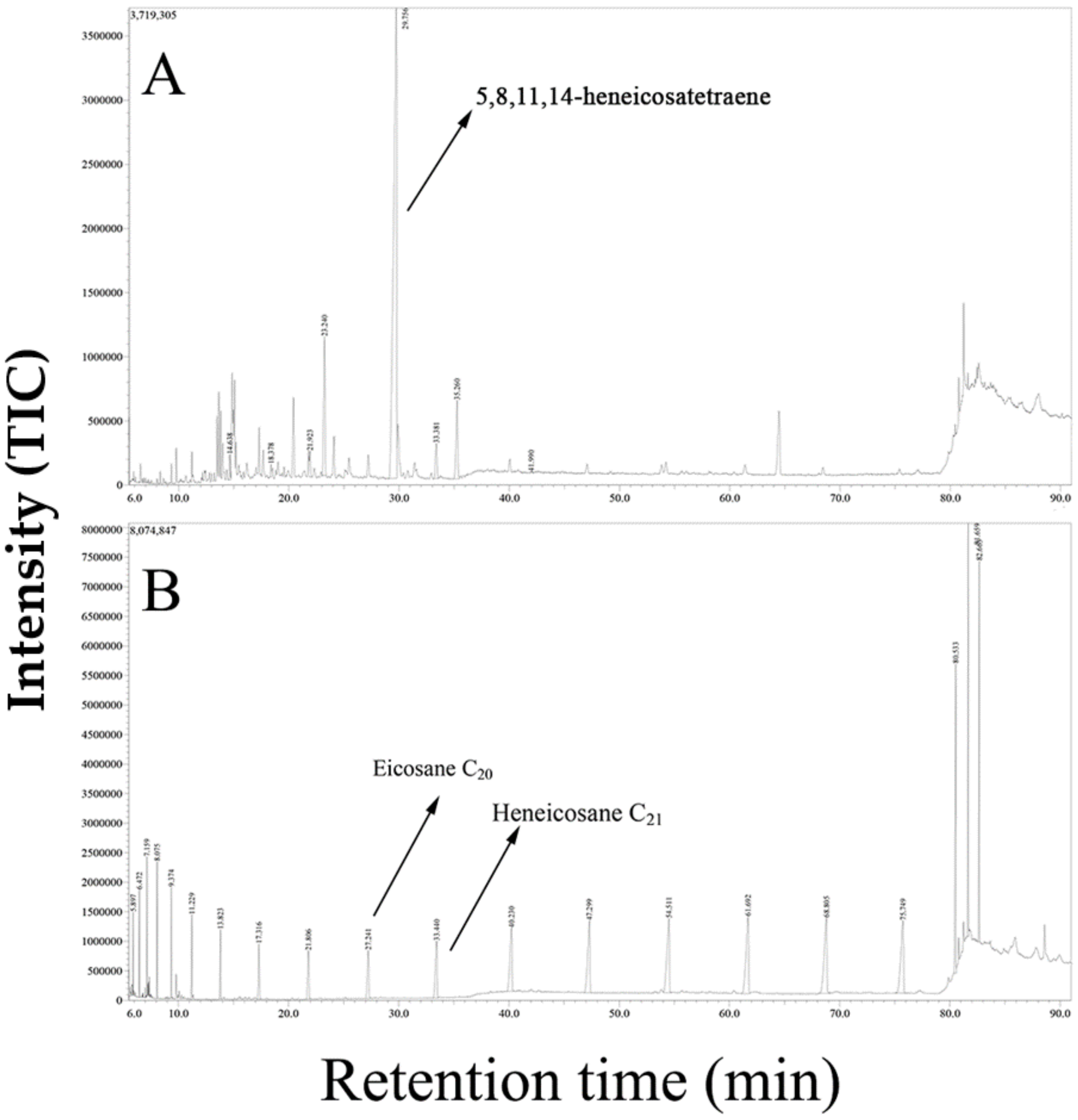
Intensity (TIC)
A
5,8,11,14-heneicosatetraene
B
Eicosane C_{20}
Heneicosane C_{21}
Retention time (min)

Figure S3 - Gas Chromatography coupled to Mass Spectrometry (GC/MS) analysis for esterification or derivatization of FA3 from *P. gymnospora*. **(A)** Arrow shows the peak for the new compound 5*Z*,8*Z*,11*Z*,14*Z*-heneicosatetraene. **(B)** Chromatogram from the hydrocarbons standards, highlighting the peaks (arrow) that eluted before eicosane (C_{20}) and after heneicosane (C_{21}). Retention times were used to calculate the retention index for 5*Z*,8*Z*,11*Z*,14*Z*-heneicosatetraene (RI = 2041).

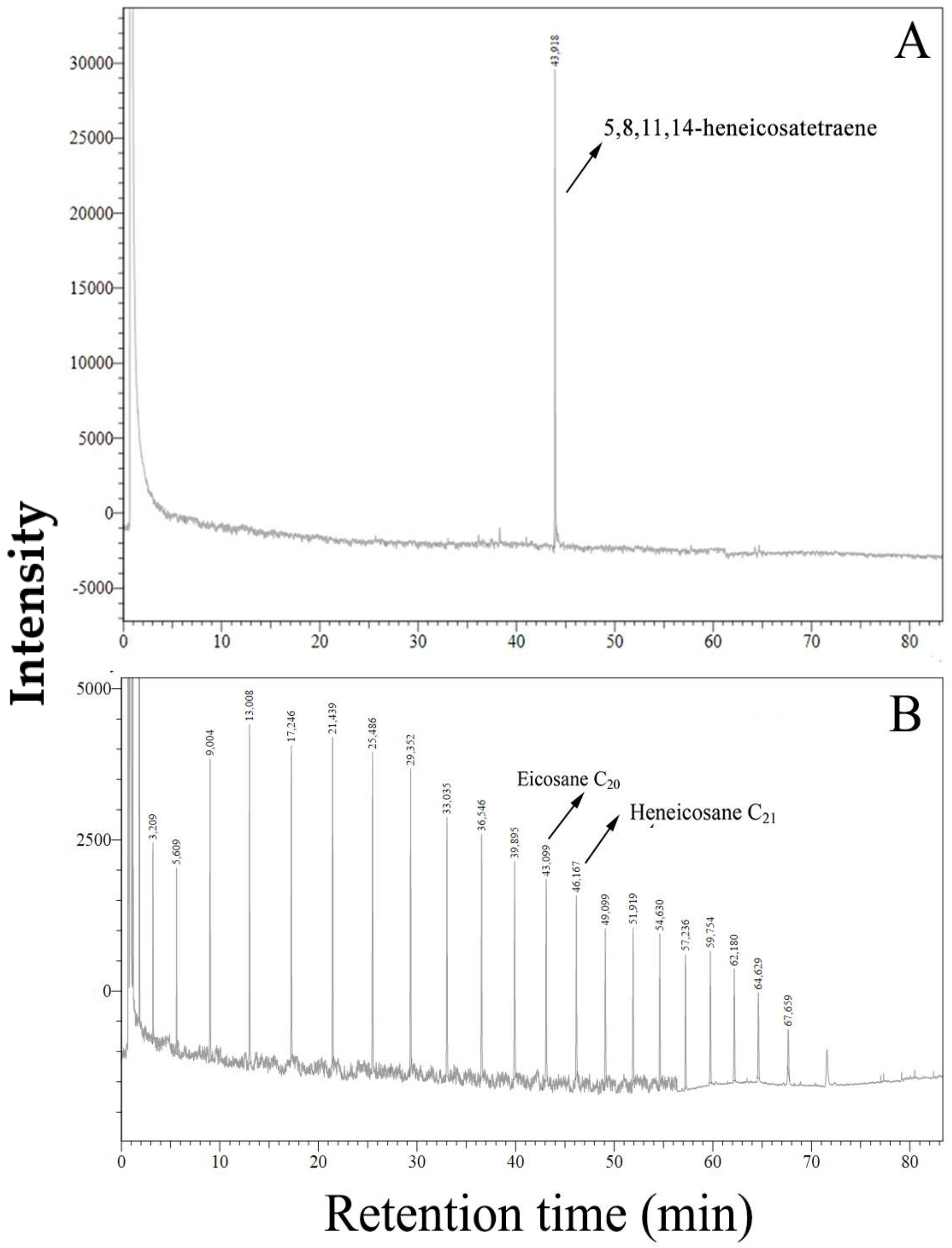

Figure S4 - Gas Chromatography coupled to Flame Ionization Detector (GC-FID) analysis without esterification of FA3 from *P. gymnospora*. **(A)** Arrow shows the peak for the new compound 5*Z*,8*Z*,11*Z*,14*Z*-heneicosatetraene. **(B)** Chromatogram from the hydrocarbons standards, highlighting the peaks (arrow) that eluted before eicosane (C_{20}) and after heneicosane (C_{21}). Retention times were used to calculate the retention index for 5*Z*,8*Z*,11*Z*,14*Z*-heneicosatetraene (RI = 2027).

Table S1. Gas Chromatography coupled to Mass Spectrometry (GC/MS) analysis of fatty acids (FAs) and sterols (STs) compounds from *P. gymnospora* isolated fractions (FA1 and FA2) and extracts (dichloromethane, DI; ethyl acetate, EA and methanol ME). X- absent chemical compounds; RT- Retention time. Results from triplicate ± standard deviation.

**Peaks**	**Compound name**	**Formula**	**Lipid number**	**Frations**				**Extracts**					
				**FA1**		**FA2**		**DI**		**EA**		**ME**	
				**RT**	**%**	**RT**	**%**	**RT**	**%**	**RT**	**%**	**RT**	**%**
1	Caprylic acid	$C_8H_{16}O_2$	8:0	X	X	X	X	X	X	11.41	0.43±0.02	6.06	1.03±0.07
2	Capric acid	$C_{10}H_{20}O_2$	10:0	X	X	8.98	0.69±0.08	X	X	13.95	0.16±0.01	8.32	0.36 ±0.03
3	Lauric acid	$C_{12}H_{24}O_2$	12:0	12.51	0.20±0.09	12.51	1.12±0.06	11.61	0.04±0.01	11.41	0.28 ±0.05	11.36	0.22 ±0.04
4	Tridecylic acid	$C_{13}H_{23}O_2$	13:0	X	X	15.42	0.20±0.04	16.14	0.04±0.01	13.95	0.32±0.05	13.88	0.07±0.02
5	Myristic acid	$C_{14}H_{23}O_2$	14:0	19.35	4.59±0.30	19.33	4.62±0.50	17.58	3.17±0.90	17.66	7.67±2.50	17.45	5.88±0.55
6	Pentadecanoic acid	$C_{15}H_{30}O_2$	15:0	24.24	0.71±0.20	24.23	0.80±0.15	21.92	0.53±0.10	21.95	3.08±1.01	21.79	1.71±0.80
7	Palmitoleic acid	$C_{16}H_{30}O_2$	16:1(n-7)	28.52	3.97±0.01	28.81	4.40±0.01	26.37	10.48±1.50	25.64	11.88±3.01	26.18	14.09±2.03
8	Palmitic acid	$C_{16}H_{32}O_2$	16:0	30.31	39.08±5.01	30.18	25.16±1.01	28.07	39.30±4.01	27.11	32.18±2.02	28.04	33.65±3.01
9	Margaric acid	$C_{17}H_{34}O_2$	17:0	36.74	0.36±0.03	X	X	33.61	0.37±0.02	33.53	1.92±0.04	33.51	0.62±0.01
10	α-linolenic acid	$C_{18}H_{30}O_2$	18:3 (n-3)	X	X	X	X	37.02	0.63±0.50	35.02	0.87±0.40	36.90	1.28±0.03
11	Linoleic acid	$C_{18}H_{32}O_2$	18:2 (n-6)	41.49	2.36±0.08	41.47	3.86±1.01	38.25	2.58±0.11	38.20	0.98±0.04	38.19	5.36±3.01
12	Oleic acid	$C_{18}H_{34}O_2$	18:1 (n-9)	42.19	38.43±5.01	42.13	52.52±4.02	39.39	23.79±2.03	39.33	18.10±3.02	39.06	14.95±3.03
13	Stearic acid	$C_{18}H_{36}O_2$	18:0	43.98	7.73±0.51	43.93	5.52±1.01	40.95	9.16±1.21	41.36	14.21±2.04	40.69	5.33±1.05
14	Arachidonic acid	$C_{20}H_{32}O_2$	20:4 (n-6)	58.95	0.79±0.05	58.91	0.45±0.06	49.86	3.28±0.51	49.83	0.92±0.02	49.72	3.15±0.51
15	Eicosapentaenoic acid	$C_{20}H_{30}O_2$	20:5(n-3)	X	X	X	X	50.29	1.55±0.05	X	X	50.11	0.89±0.04
16	Di-homo-γ-linolenic acid	$C_{20}H_{34}O_2$	20:3 (n−6)	X	X	X	X	X	X	X	X	51.07	1.61±0.03
17	Eicosadienoic acid	$C_{20}H_{36}O_2$	20:2 (n-6)	X	X	X	X	52.57	0.19±0.01	X	X	X	X
18	Gondoic acid	$C_{20}H_{38}O_2$	20:1(n-9)	X	X	X	X	52.98	0.26±0.02	X	X	X	X
19	Eicosanoic acid SD	$C_{20}H_{40}O_2$	20:0	X	X	X	X	55.22	2.47±2.01	55.52	5.44±1.02	55.52	5.44±0.51
20	Erucic acid	$C_{22}H_{42}O_2$	22:1(n-9)	X	X	X	X	67.86	1.35±0.61	X	X	67.76	1.16±0.53
21	Behenic acid	$C_{22}H_{44}O_2$	22:0	X	X	X	X	69.84	0.32±0.05	70.00	1.37±0.31	69.77	0.33±0.02
22	Tricosylic acid	$C_{23}H_{46}O_2$	23:0	X	X	X	X	X	X	77.12	0.19±0.02	X	X

22	Lignoceric acid	$C_{24}H_{48}O_2$	24:0	81.74	0.25±0.03	81.74	0.20±0.01	80.92	0.36±0.02	X	X	80.95	0.86±0.06
23	Cerotic acid	$C_{26}H_{52}O_2$	26:0	X	X	X	X	83.35	0.13±0.01	83.42	0.87±0.05	83.42	1.20±0.03

Table S2 - PERMANOVA results of the overall test of treatments level differences. Analysis assumes the factor class is fixed and uses III sums of squares. Significance (*< 0.05).

Source	df	SS	MS	Pseudo-F	Unique perms
Treatments	5	18720	3744	123,16	999
Residual	12	364,81	30,401		
Total	17	19085			

Table S3 - PERMANOVA Results of the *post-hoc* pairwise tests, showing the *t*-statistic, number of unique permutations in the procedure (significance, *< 0.05). Bray Curtis similarity of FAs derivatives composition of treatments (n=3) with six fixed levels (dichloromethane DI; ethyl acetate EA; methanol ME, fatty acid FA1, fatty acid FA2 and fatty acid FA3).

**Groups**	***t***	**Unique perms**	***P* (perm)**
Dichloromethane, ethyl acetate	3.8307	10	0.1040
Dichloromethane, methanol	3.5754	10	0.1090
Dichloromethane, fatty acid FA1	3.8566	10	0.1056
Dichloromethane, fatty acid FA2	7.9251	10	0.1014
Dichloromethane, fatty acid FA3	18.8930	10	*0.0014
Ethyl acetate, methanol	3.2504	10	0.1128
Ethyl acetate, fatty acid FA1	6.0152	10	0.0988
Ethyl acetate, fatty acid FA2	8.9469	10	0.0972
Ethyl acetate, fatty acid FA3	18.1980	10	*0.0013
Methanol, fatty acid FA1	6.3041	10	0.0920
Methanol, fatty acid FA2	8.7372	10	0.9940
Methanol, fatty acid FA3	17.753	10	*0.0024
Fatty acid FA1, fatty acid FA2	4.3973	10	0.1034
Fatty acid FA1, fatty acid FA3	20.335	10	*0.0001
Fatty acid FA2, fatty acid FA3	25.4900	10	*0.0001

Table S4 - PERMANOVA results of average similarity within groups (dichloromethane DI; ethyl acetate EA; methanol ME, fatty acid FA1, fatty acid FA2 and fatty acid FA3).

	DI	EA	ME	FA1	FA2	FA3
DI	91.854					
EA	79.168	91.460				
ME	79.897	81.122	90.979			
FA1	80.596	69.909	67.551	92.334		
FA2	66.063	60.514	59.868	82.182	94.665	
FA3	23.184	23.255	21.891	23.44	22.001	95.381

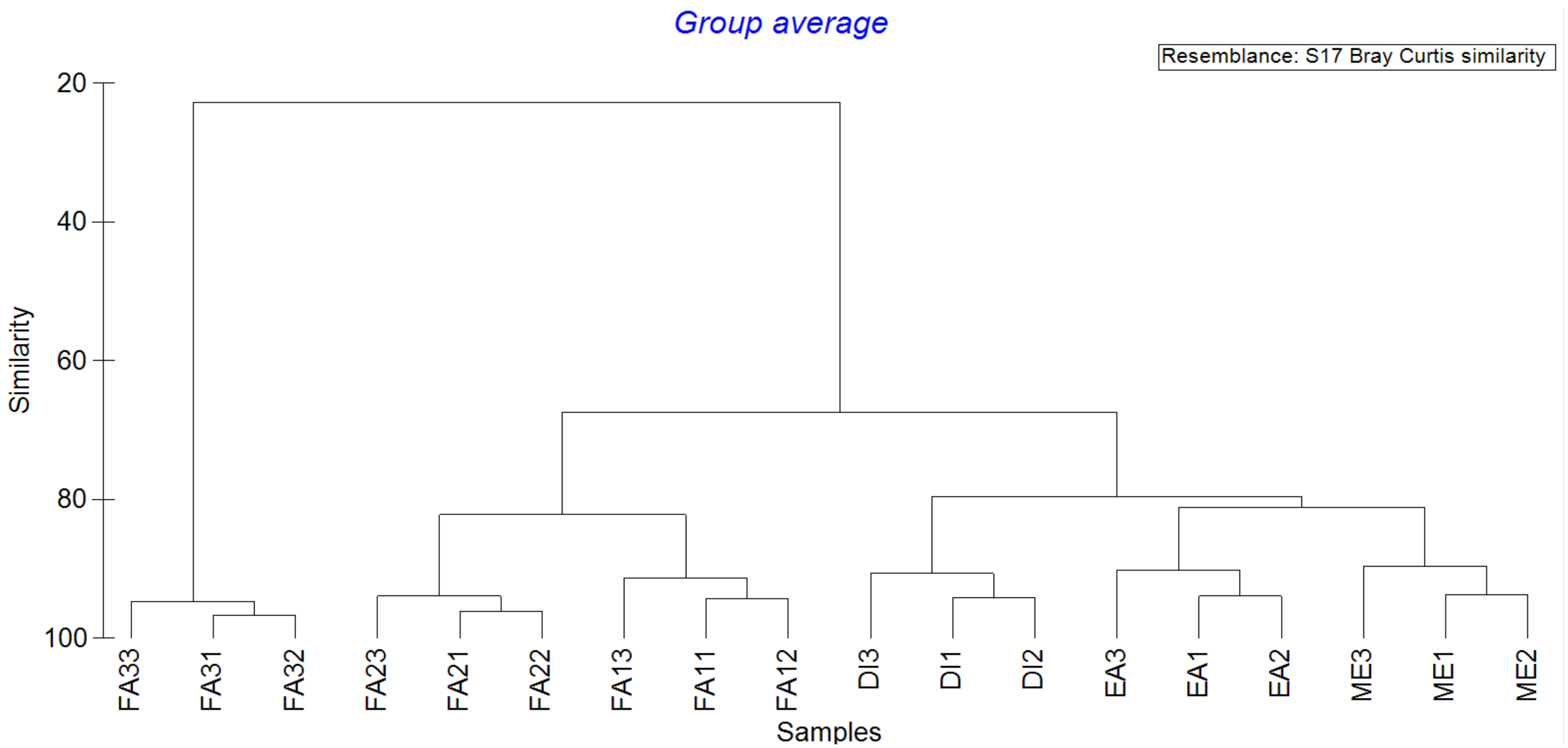

Figure S5. Cluster analyses (Bray Curtis similarity) based on FAs derivatives composition according to GC/MS of *P. gymnospora* extracts and fractions (DI, EA, ME, FA1 and FA2 from FA3).
